# The effects of music listening on somatic symptoms and stress markers in the everyday life of women with somatic complaints and depression

**DOI:** 10.1038/s41598-021-03374-w

**Published:** 2021-12-15

**Authors:** Anja C. Feneberg, Ricarda Mewes, Johanna M. Doerr, Urs M. Nater

**Affiliations:** 1 Department of Clinical and Health Psychology, Faculty of Psychology, University of Vienna, Vienna, Austria; 2 University Research Platform “The Stress of Life (SOLE) – Processes and Mechanisms underlying Everyday Life Stress”, University of Vienna, Vienna, Austria; 3 Outpatient Unit for Research, Teaching and Practice, Faculty of Psychology, University of Vienna, Vienna, Austria; 4 Department of Neurology, University Hospital Giessen and Marburg, Giessen, Germany

**Keywords:** Biomarkers, Signs and symptoms, Psychology, Human behaviour

## Abstract

Despite a growing body of literature documenting the health-beneficial effects of music, empirical research on the effects of music listening in individuals with psychosomatic disorders is scarce. Using an ambulatory assessment design, we tested whether music listening predicts changes in somatic symptoms, subjective, and biological stress levels, and examined potential mediating processes, in the everyday life of 58 women (M = 27.7 years) with somatic symptom disorder (SSD) and depressive disorders (DEP). Multilevel models revealed that music listening predicted lower subjective stress ratings (*p* ≤ 0.02) irrespective of mental health condition, which, in turn, predicted lower somatic symptoms (*p* ≤ 0.03). Moreover, specific music characteristics modulated somatic symptoms (*p* = 0.01) and autonomic activity (*p* = 0.03). These findings suggest that music listening might mitigate somatic symptoms predominantly via a reduction in subjective stress in women with SSD and DEP and further inform the development of targeted music interventions applicable in everyday life.

## Introduction

Somatic symptoms (e.g., headache, back pain, fatigue, bloating), which are often not fully explained by an organic pathology (i.e., medically unexplained), are common in daily life. In a survey conducted in Germany, around 80% of the general population reported at least one mildly impairing symptom during the past week^1^. For some individuals, e.g., persons with somatic symptom disorder (SSD) or a depressive disorder, such symptoms may persist over months or years and become extremely debilitating. Individuals with SSD suffer from at least one persistent somatic symptom related to excessive thoughts, health anxiety, and/or time devoted to the symptom(s), resulting in significant social and functional restrictions in daily life. It has an estimated point prevalence of 5.4%^2^. Depressive disorders (DEP) are primarily characterized by dysfunctional affect regulation. However, around two thirds of patients with DEP in primary care settings initially present with painful and non-painful somatic symptoms^3^ that might negatively impact disease trajectories and should therefore be addressed by specific interventions^4^. Although the biological underpinnings of SSD and DEP are not yet fully unravelled^5^, the current literature emphasizes the role of chronic stress and dysregulations in the stress-responsive systems, the hypothalamic–pituitary–adrenal (HPA) axis and the autonomic nervous system(ANS), in the development and exacerbation of both mental disorders^6,7^. Importantly, alterations in HPA axis activity seem to be disorder-specific: while the evidence indicates that hypocortisolism is a risk factor for conditions characterized by medically unexplained somatic symptoms^7,8^, HPA hyperactivity seems to be strongly associated with DEP^9^. ANS dysfunctions related to sympathetic predominance are discussed for both disorders^10,11^. Acute perceived stress and heightened physiological arousal are, in turn, assumed to aggravate the perception of somatic symptoms^12^. Moreover, we found that lower cortisol levels were prospectivly associated with higher intensity of somatic symptoms in SSD in the same sample as used in the present study^13^. Consequently, HPA axis/ANS dysfunctions can qualify as potential treatment targets in individuals with SSD and DEP^14^. In this regard, interventions should aim at harmonizing dysregulated stress-system activity patterns^15,16^, with different implications for both disorders: while a downregulation of cortisol secretion should be aimed for in patients with DEP, the opposite seems to be favorable for individuals with SSD.

Available treatment options for the management of somatic symptoms such as cognitive-behavioral therapies are not accepted by some patients and only yield small to medium effect sizes^17,18^, and pharmacological agents might cause negative side-effects, with a risk of further increasing somatic complaints^19^. Hence, there is a great need for complementary treatments that can be easily self-administered by individuals suffering from SSD and DEP. Music can be seen as a promising tool for symptom alleviation, considering its documented benefits for pain reduction^20^, affect regulation^21^ and biological stress systems^22,23^. Furthermore, there is ample evidence on the beneficial effects of music for reducing depressive symptoms in patient populations^24,25^. However, empirical studies on the effects of music on somatic symptoms in either SSD or DEP is extremely scarce and inconclusive. Existing systematic reviews and meta-analyses indicate that music interventions reduced pain and fatigue with small to medium effect sizes in patients with cancer and functional somatic syndromes (e.g., fibromyalgia, irritable bowel syndrome)^26,27^. Although these patient populations differ from SSD and DEP in terms of their pathophysiology and symptom trajectories, these findings support the notion that music might have the capacity to alleviate somatic symptoms in individuals with SSD and DEP as well.

Ambulatory assessment methods are increasingly being implemented in clinical research, enabling the assessment of behaviors and symptoms as they occur and fluctuate in daily life^28^. These approaches are ideally suited to examine psychobiological microprocesses unfolding in relation to music listening with the advantages of minimal recall bias and maximal ecological validity^29^. Besides previous ambulatory assessment studies indicating stress-reducing effects of music listening in healthy individuals^30–32^, comparable investigations focusing on the beneficial effects of music listening in individuals with somatic symptoms are lacking. One exception to this is a previous study from our own lab, where we examined the effects of music listening on ratings of pain and stress markers in the everyday life of 30 women suffering from fibromyalgia, a chronic pain condition^33^. Interestingly, in particular, music that was perceived as high in valence (ranging from sad to happy) had a positive impact on pain ratings. Other studies conducted in experimental lab-based and clinical settings also found that high valence and low arousal of music (i.e., ‘relaxing music’) decreased pain and stress markers^34–36^. In addition, previous ambulatory assessment studies documented that reasons for music listening (i.e., motivations to engage in music listening) such as ‘relaxation’ and ‘activation’ predicted lower levels of pain and stress^30,32,33^. Consequently, characteristics of the music and reasons for music listening might be important modulators in the context of music listening in everyday life^30^, although their impact on somatic symptoms and stress markers in individuals with SSD and DEP has not been systematically investigated.

Considering potential mechanisms of how music might exert its health-beneficial effects, previous research has advocated the biopsychological mediation model of music listening^23,37^, which posits that stress-responsive systems may act as mediating agents in the relationship between music listening and somatic symptoms. Importantly, previous studies support the notion that the processing of music involves brain areas that are responsible for the regulation of the body’s stress systems^38^. For instance, it is assumed that an early processing of acoustical features (e.g., tempo, loudness, pitch) in the brainstem contributes to changes in autonomic markers, such as heart and breathing rate, via autonomic reflexes^39^. Furthermore, limbic regions including the hypothalamus and the amygdala are involved in the emotional processing of music. Importantly, these regions also play key roles in governing neuroendocrine and autonomic functioning and in the affective-motivational modulation of somatic complaints^40^. Thus, music-induced changes in the central nervous system may lead to altered biological stress levels and states of relaxation, which might further alleviate the perceived intensity of and impairment by somatic symptoms. Nonetheless, the mediating role of the stress-responsive systems in the relationship between music and somatic symptoms has not yet been empirically tested.

### The present study

In the context of a larger research project^13^, the present study sought to unravel the differential effects of music listening on somatic symptoms as well as on subjective and biological stress markers in the everyday life of women with SSD and DEP. While music listening might be similarly related to lower somatic symptoms and ANS activity in SSD and DEP, the influence of music listening on HPA axis activity might differ, due to the above-mentioned differential HPA axis dysfunctions in both disorders. Indeed, listening to relaxing music was associated with an increase in cortisol secretion in a mixed sample of psychosomatic inpatients (most of which were diagnosed with somatoform disorder)^35^. However, the potentially differential effects of music listening on HPA axis activity in individuals with SSD vs. DEP have not yet been systematically examined. Furthermore, we aimed to elucidate biopsychological mechanisms underlying the relationship between music listening and somatic symptoms. We hypothesized that:

1. Music listening predicts lower intensity of and impairment by somatic symptoms irrespective of mental health condition (SSD/DEP). Moreover, music that is perceived as high in valence (ranging from sad to happy) and low in arousal (ranging from calming to energizing) is associated with lower intensity of and impairment by somatic symptoms.
2. Music listening predicts lower ratings of subjective stress and ANS activity (indicated by salivary alpha-amylase) in both conditions. In addition, music listening predicts higher HPA axis activity (indicated by salivary cortisol) in individuals with SSD and lower HPA axis activity in individuals with DEP. Moreover, for both conditions, music that is perceived as high in valence is associated with lower levels of subjective stress and music that is perceived as low in arousal is associated with lower levels of subjective stress and ANS activity.
3. Subjective and biological stress markers mediate the relationship between music listening and intensity of and impairment by somatic symptoms.

In addition, we aimed at exploring the role of reasons for music listening in reducing levels of somatic symptoms and stress in both individuals with SSD and DEP.

## Results

### Descriptive analyses

#### Participant characteristics

The final sample consisted of 29 participants with SSD and 29 participants with DEP (M = 27.7 ± 10.1 years of age). The SSD and DEP groups did not differ significantly with respect to somatic symptoms as assessed via the Patient Health Questionnaire (PHQ-15), while the DEP group scored significantly higher on depressive symptoms (assessed via the PHQ-9). Further details on demographic and clinical characteristics are presented in Table 1.

**Table 1:** Demographic and clinical characteristics by mental health condition.

	SSD	DEP	Test parameter (df)	*p*
	N = 29	N = 29		
Age (years, mean ± SD; range)	29.8 ± 12.7; 19–64	25.6 ± 6.2; 19–51	*t* = 1.6 (56)	0.11
**Highest educational level (n, %)**			*χ*^*2*^ = 4.7 (2)	0.10
Medium-track secondary school	4 (13.8%)	–		
Advanced technical college entrance qualification	4 (13.8%)	3 (10.3%)		
University entrance level	21 (72.4%)	26 (89.7%)		
**Monthly income (in Euro)**^a^ (n, %)			*χ*^*2*^ = 2.2 (2)	0.33
< 1250	20 (69.0%)	23 (82.1%)		
1250–2999	6 (20.7%)	2 (7.2%)		
3000–5000	3 (10.3%)	3 (10.7%)		
BMI (kg/m^2^, mean ± SD; range)	22.3 ± 3.1; 17.3–29.1	22.3 ± 2.9; 18.4–29.0	*t* = 0.1 (56)	0.94
PHQ-15 (mean ± SD)	9.4 ± 3.0	8.0 ± 3.9	*t* = 1.6 (56)	0.11
PHQ-9 (mean ± SD)	5.9 ± 3.0	15.9 ± 4.2	*t* = − 10.4 (56)	** < 0.001**
Intake of pain medication (n, %)	8 (27.6%)	1 (3.4%)	*χ*^*2*^ = 6.4 (1)	**0.011**
Intake of antidepressant medication (n, %)	1 (3.4%)	4 (13.8%)	*χ*^*2*^ = 2.0 (1)	0.16
**Duration of somatic complaints**^a^ (n, %)			*χ*^*2*^ = 11.4 (3)	**0.010**
< 1 year	–	7 (24.1%)		
1 year	7 (24.1%)	6 (20.7%)		
> 1 year	22 (75.9%)	13 (44.8%)		
Life-long	–	2 (6.9%)		

*DEP* depressive disorders, *SSD* somatic symptom disorder, *BMI* Body Mass Index, *PHQ-15* Patient Health Questionnaire, somatic symptoms subscale, excluding two items from the PHQ-9 scale, *PHQ-9* Patient Health Questionnaire, depressive symptoms subscale. Scores ≥ 5, ≥ 10, and ≥ 15 indicate low, medium, and high symptom severity on the (original) PHQ-15 and PHQ-9 scales.

^a^information unavailable for one individual in the DEP group. Significant estimates (*p* < 0.05) are marked in bold.

Participants’ reports on the Music Preference Questionnaire (revised version) regarding habitual music behaviors revealed that the two groups considered music to be of equal and rather high importance in their lives (SSD group: 4.3 ± 0.8; DEP group: 4.3 ± 0.9; *p* > 0.99). Individuals in the SSD group reported listening to music for an average of 97.6 ± 87.3 minutes and individuals in the DEP group reported listening to music for 141.4 ± 120.2 minutes per day (n.s., *p* = 0.13). Both groups did not noticeably differ regarding other aspects of habitual music behavior. Details are reported in Table S1 in the Supplemental Material available online.

#### Ambulatory assessment

Concerning music listening in daily life, an average of 10.4 ± 7.0 music episodes (range 0–31) was reported per participant throughout the 14-day ambulatory assessment period. One participant reported no music listening at all. Overall, the SSD group reported 273 music listening episodes (14.8% of observations), with musical valence being rated as rather happy (M = 74.2 ± 23.5) and musical arousal being rated as rather energizing (M = 60.3 ± 31.9). The DEP group reported 321 music listening episodes (18.1% of observations), with musical valence also rated as rather happy (M = 65.8 ± 25.5) and musical arousal rated as rather energizing (M = 59.9 ± 28.0). ‘Activation’ was the most commonly stated reason for music listening (SSD 42.1%, DEP 36.4%), followed by ‘relaxation’ (SSD 30%, DEP 26.2%), ‘distraction’ (SSD 19.8%, DEP 25.9%), ‘reducing boredom’ (SSD 12.5%, DEP 26.2%), and ‘no reason’ (SSD 14.3%, DEP 17.1%). Descriptive statistics for somatic symptoms and stress markers are reported in Table 2.

**Table 2:** Descriptive statistics of somatic symptoms and stress markers by mental health condition.

	SSD	DEP	Test parameter (df)	*p*	ICC
	M ± SD (n)	M ± SD (n)			
Intensity of somatic symptoms^a^	26.8 ± 23.9 (1848)	15.0 ± 20.1 (1773)	*t* = 16.1 (3619)	** < 0.001**	0.44
Impairment by somatic symptoms﻿^a^	24.6 ± 26.1 (1848)	16.2 ± 23.4 (1773)	*t* = 10.1 (3619)	** < 0.001**	0.36
Subjective stress^b^	1.5 ± 1.0 (1848)	2.0 ± 1.1 (1773)	*t* = − 12.9 (3619)	** < 0.001**	0.27
Salivary cortisol (nmol/l)^c^	4.9 ± 5.3 (1792)	4.7 ± 5.6 (1711)	*t* = 3.4 (3501)	**0.001**	0.18
Salivary alpha-amylase (U/min)^c,d^	39.0 ± 45.6 (1713)	38.0 ± 44.2 (1648)	*t* = 1.1 (3359)	0.28	0.49

Values are averages across individuals and across measurement time points. *DEP* depressive disorders, *SSD* somatic symptom disorder, *ICC* intraclass correlation coefficient.

^a^Assessed on a visual analog scale ranging from 0 (‘not at all’) to 100 (‘strongest imaginable’/‘very much’).

^b^Assessed on a 5-point Likert scale ranging from 0 (‘not at all’) to 4 (‘very much’).

^c^Group comparisons based on transformed values (ln(*x*) + 10).

^d^Corrected for salivary flow rate. Significant estimates (*p* < 0.05) are marked in bold.

### Hypothesis 1: effects of music listening on somatic symptoms

Music listening per se did not predict momentary intensity of somatic symptoms (UC = 0.11, *p* = 0.91) or impairment by somatic symptoms (UC = − 1.2, *p* = 0.30). However, when investigating perceived music characteristics, musical valence predicted lower intensity of and impairment by somatic symptoms, as reported in Table 3. Thus, music perceived as happier (relative to an individual’s average reports) predicted lower intensity of and impairment by somatic symptoms at the subsequent time point, while music perceived as sadder predicted higher intensity of and impairment by somatic symptoms. Arousal of music did not predict intensity of or impairment by somatic symptoms. Music characteristics explained 4.1% of the residual within-person variance in intensity of somatic symptoms (*χ*^*2*^(7) = 15.75, *p* = 0.027) and 4.2% of the residual within-person variance in impairment by somatic symptoms (*χ*^*2*^(7) = 21.45, *p* = 0.004). None of the cross-level interactions with mental health condition were significant, indicating that the associations did not differ significantly between the SSD and DEP group (all UC ≤ 0.05, *p* ≥ 0.45).

**Table 3:** Multilevel models for somatic symptoms predicted by music characteristics and covariates.

*Fixed effects*	Model 1a) Intensity of somatic symptoms				Model 1b) Impairment by somatic symptoms			
	UC	SE	df	*p*	UC	SE	df	*p*
**Level 2**								
Intercept level 2	**12.92**	**4.23**	**48**	**0.004**	**15.56**	**4.31**	**48**	** < 0.001**
PHQ-15	**1.95**	**0.54**	**48**	** < 0.001**	**1.96**	**0.52**	**48**	** < 0.001**
**Level 1**								
Musical valence	** − 0.14**	**0.05**	**56**	**0.012**	** − 0.14**	**0.06**	**56**	**0.012**
Musical arousal	− 0.00	0.04	56	0.96	0.00	0.04	56	0.84
Medication intake	**15.20**	**3.54**	**413**	** < 0.001**	**20.85**	**3.87**	**413**	** < 0.001**

*Random effects*	VC	SD	*χ*^*2*^ (df)	*p*	VC	SD	χ2 (df)	*p*
Intercept level 1	**137.23**	**11.72**	**220.21 (39)**	** < 0.001**	**152.72**	**12.36**	**214.77 (39)**	** < 0.001**
Musical valence	0.02	0.14	54.14 (47)	0.22	0.01	0.12	61.16 (47)	0.080
Musical arousal	0.00	0.02	33.75 (47)	> 0.50	0.00	0.07	51.42 (47)	0.30
Residual	287.17	16.95			344.86	18.57		

Only significant covariates are displayed. Complete results are reported in Table S2 in the Supplemental Material available online. *UC* unstandardized coefficient, *SE* standard error, *df* degrees of freedom, *VC* variance component, *SD* standard deviation, *PHQ-15* somatic symptom severity subscale from the Patient Health Questionnaire, Significant estimates (*p* < 0.05) are marked in bold.

### Hypothesis 2: effects of music listening on stress markers

#### Subjective stress

Music listening per se predicted lower levels of subjective stress ratings (UC = − 0.12, *p* = 0.018) and explained 0.5% of the residual within-person variance in subjective stress (*χ*^*2*^(3) = 11.29, *p* = 0.010). This effect did not depend on mental health condition, as indicated by the non-significant cross-level interaction (UC = -0.03, *p* = 0.80). Neither valence (UC = − 0.00, *p* = 0.087) nor arousal (UC = − 0.00, *p* = 0.70) of music predicted subjective stress levels, and cross-level interactions were not significant (all UC < 0.01, *p* ≥ 0.72).

#### Salivary cortisol (sCort)

Music listening did not predict sCort levels (UC = -0.01, *p* = 0.75). Moreover, music characteristics did not predict sCort levels (all UC < 0.00, *p* ≥ 0.21), and none of the cross-level interactions were significant (all UC ≤ 0.05, *p* ≥ 0.32).

#### Salivary alpha-amylase (sAA)

Music listening per se did not predict sAA output (UC = 0.06, *p* = 0.26). Concerning music characteristics, musical valence did not predict sAA output (UC = − 0.00, *p* = 0.068), but musical arousal predicted higher sAA output (UC = 0.004, *p* = 0.025), irrespective of mental health condition (UC = 0.00, *p* = 0.93). Thus, music perceived as more energizing (relative to an individual’s average reports) predicted higher sAA output at the subsequent time point, while music perceived as more calming predicted lower sAA output. Music characteristics explained 3% of the residual within-person variance in sAA output (*χ*^*2*^(7) = 8.50, *p* = 0.28). We additionally conducted all analyses with sAA activity levels uncorrected for salivary flow rate as outcome. Overall, the same pattern of results emerged, except for the finding that musical valence additionally predicted lower sAA activity levels.

### H﻿ypothesis 3: mediation via stress markers

The previous analyses revealed that, irrespective of mental health condition, music listening predicted lower subjective stress ratings and musical arousal predicted sAA output. Hence, we examined 1–1–1 multilevel mediation models focusing on these specific (music-related) predictors and (stress-related) mediators with somatic symptoms as outcomes, combining data from both groups.

**Figure 1:**
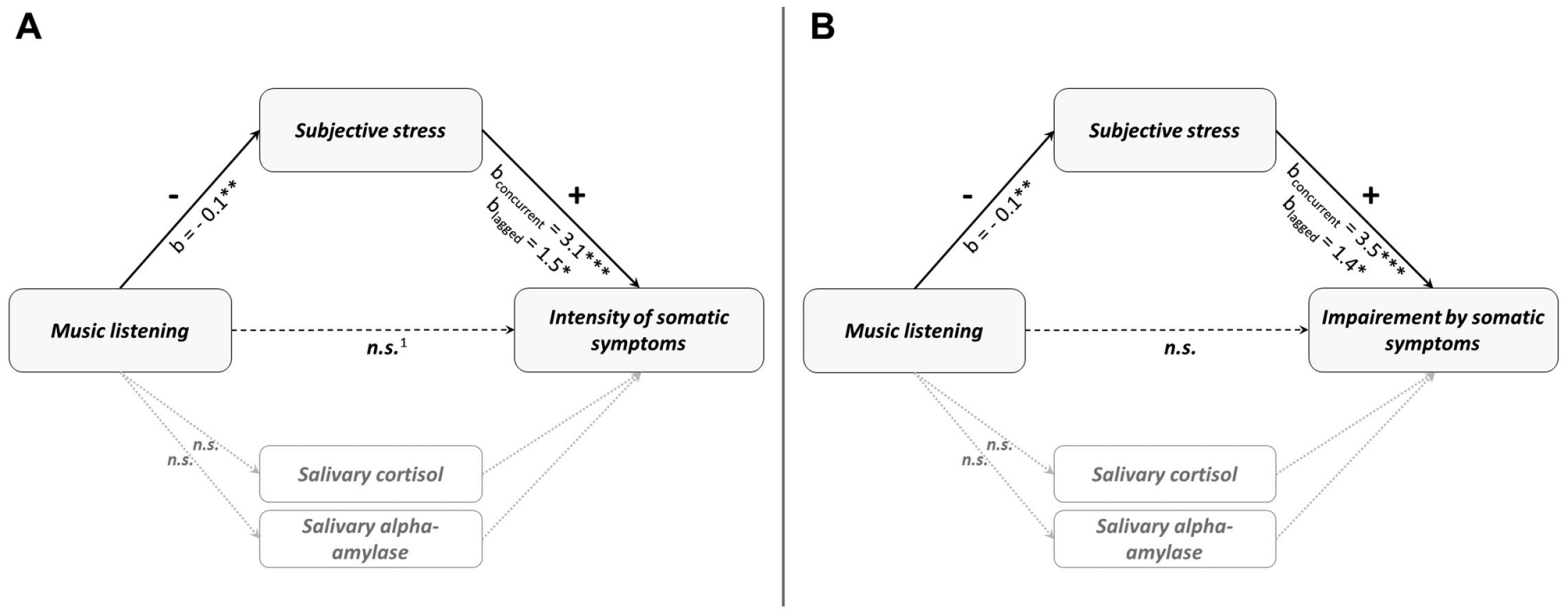
Lower-level mediation models (1–1–1) for the effect of music listening on somatic symptoms via subjective stress. Music listening temporarily preceded subjective stress (mediator) and somatic symptoms (outcome). Both concurrent and time-lagged effects between mediator and outcome were computed. As music listening did not predict salivary stress markers (cortisol, alpha-amylase), no mediation analyses were computed with these parameters. (**A**) Mediation model for (concurrent/time-lagged) intensity of somatic symptoms. (**B**) Mediation model for (concurrent/time-lagged) impairment by somatic symptoms. ^1^b = 2.0* for lagged intensity of somatic symptoms. *p < 0.05, **p < 0.01, ***p < 0.001.

As shown in Fig. 1, multilevel mediation models including the most relevant covariates (medication intake, time since awakening, sleep quality, PHQ-15 based on the previous analyses) revealed a significant negative indirect effect of having listened to music on momentary intensity of (Est. = − 0.36, 95% MCCI [− 0.79; − 0.09]) and impairment by (Est. =  − 0.41, 95% MCCI [− 0.78; − 0.10]) somatic symptoms via concurrently reported momentary subjective stress on the within-person level. Furthermore, we conducted analyses considering a time distance between the assessment of subjective stress and the assessment of somatic symptoms. These analyses revealed significant indirect effects on the within-person level of having listened to music via momentary subjective stress on lagged reports of intensity of (Est. =  − 0.18, 95% MCCI [− 0.41; − 0.02]) and impairment by (Est. =  − 0.17, 95% MCCI [− 0.39; − 0.01]) somatic symptoms. Indirect effects on the between-person level were not significant (all Est. ≤ 1.31, 95% MCCI [− 7.29; 11.59]) and none of the indirect effects with sAA output as a mediator were significant (all Est. ≤ 0.00, 95% MCCI [− 0.07; 0.05]).

### Exploratory analyses: role of reasons for music listening

None of the reasons for music listening predicted intensity of or impairment by somatic symptoms (all UC ≤ 3.50, *p* ≥ 0.19).

Furthermore, there were no significant main effects of reasons for music listening on subjective stress ratings (all UC ≤ 0.14, *p* ≥ 0.25). However, when including cross-level interactions (reasons x mental health condition), the analyses revealed significant interaction effects for ‘activation’ (UC = 0.66, *p* = 0.020), ‘distraction’ (UC = 0.61, *p* = 0.017), and ‘no reason’ (UC = 0.92, *p* = 0.046), indicating higher subjective stress ratings in women with SSD compared to women with DEP when listening to music for these reasons. Moreover, listening to music for ‘no reason’ predicted lower levels of subjective stress in women with DEP (UC = -0.56, *p* = 0.040). The model including cross-level interactions explained 7.5% of the residual within-person variance in subjective stress ratings compared to the control model (*χ*^*2*^(30) = 24.25, *p* > 0.50). Results of the full model are presented in Table S3 in the Supplemental Material available online.

Considering biological stress markers, having listened to music for ‘no reason’ predicted lower sAA output (UC = − 0.29, *p* = 0.029), irrespective of mental health condition (UC = − 0.01, *p* = 0.98). The other reasons did not predict sAA output (all UC < 0.00, *p* > 0.10). Reasons for music listening explained 8.5% of the residual within-person variance in sAA output (*χ*^*2*^(25) = 19.60, *p* > 0.50). Reasons for music listening did not predict sCort levels (all UC ≥ − 0.17, *p* ≥ 0.16) and no cross-level interactions were significant (all UC ≤ 0.35, *p* ≥ 0.12).

## Discussion

The present study revealed beneficial effects of music listening in the everyday life of women with SSD and DEP, with three main findings: First, not music listening per se, but rather music characteristics, seemed to be relevant for the mitigation of somatic symptoms and downregulation of ANS activity, irrespective of mental health condition. Second, while HPA axis activity remained unaffected by music listening in both groups, music listening predicted lower levels of subjective stress. In this regard, why one listened to music seemed to matter, since differential associations were found for individuals in the SSD and DEP group. Third, subjective stress (but not biological stress markers) mediated the effect of music on somatic symptoms.

These results replicate and extend findings from previous studies. Remarkably, similar studies with healthy individuals reported music listening in around 25–40% of sampled reports^31,41^. In the present study, individuals with DEP recorded around 18% of music listening episodes, while participants with SSD reported music listening in only 15% of the sampled reports. As music was highly liked by both groups, this raises the question whether (and why) individuals with certain health conditions do not use music listening as a self-management tool in their daily life as frequently as healthy individuals do. Considering the beneficial effects regarding symptom relief and stress reduction that were revealed in this and previous studies^22,26,33,40^, raising more awareness among individuals suffering from somatic symptoms about the potential benefits of self-administered music listening in daily life could be advantageous.

Although having listened to music per se was not associated with lower somatic symptoms, we were able to demonstrate that when individuals engaged in music listening, particularly music perceived as happy, irrespective of its arousal, predicted lower levels of somatic symptoms in both SSD and DEP. There are several possible explanations for this finding. Particularly with regard to painful somatic complaints, there is evidence that pleasurable and happy music has the capacity to evoke acitivity in the descending pain-modulatory system, a phenomenon known as ‘music-induced analgesia’^42^. Furthermore, recent EMA studies indicate that positive affect is more strongly associated with lower somatic symptoms than negative affect (inversely) on the within-person level^43,44^. Consequently, particularly music perceived as happy might induce positive affective states that modulate symptom perception. Additionally, happy music might have supported shifts of attention away from somatic symptoms and towards more favorable (internal/external) stimuli. Concerning musical arousal, the available evidence seems to be inconsistent. Calming music has been frequently used as a stimulus to successfully attenuate somatic symptoms in previous research (e.g.,^35,45^), but only few studies actually controlled for musical valence. In agreement with our finding, studies that examined the role of musical valence and musical arousal concurrently indicate that the arousal of the music seems to be of minor relevance regarding the modulation of somatic symptoms^33,46^.

As expected, musical arousal predicted sAA output, with energizing music being related to an upregulation and relaxing music being related to a downregulation of ANS activity. The responsivity of the ANS to features determining musical arousal (e.g., timbre, loudness) has been shown in a plethora of previous studies (for an overview see^47^), and the same pattern of results was found in previous ambulatory assessment studies with healthy individuals^30,48^. Thus, our result suggests that in individuals with DEP and SSD, the ANS is responsive to musical stimulation and reacts in the expected direction depending on musical arousal.

Contrary to our hypotheses, we did not find any changes in cortisol concentrations in SSD or DEP after music listening. Interestingly, although music listening for the purpose of ‘relaxation’ was associated with lower cortisol levels in the daily life of healthy individuals^30^, there was no association between music listening and HPA activity in our previous ambulatory assessment study with fibromyalgia patients^33^. Thus, the present study could substantiate the notion that mere music listening in daily life might not be sufficiently powerful to affect cortisol levels in populations known to suffer from HPA dysregulations^30^. Future research in individuals with stress-associated disorders should consider investigating more intense music engagements (e.g., choir singing, more listening, or listening to music with others), which might reveal a stronger impact on HPA axis activity^48^.

Furthermore, our results suggest that mere music listening in everyday life predicts lower levels of subjective stress in both SSD and DEP. While characteristics of the music seemed to be of minor importance in this regard, ‘distraction’, ‘acitvation’, and ‘no reason’ modulated this effect depending on mental health condition. Regarding music characteristics, in contrast to our findings, Sandstrom and Russo^34^ emphasized the role of musical valence and arousal in reducing subjective stress levels. However, their study was undertaken in a lab-based context applying researcher-selected music. Our findings are broadly consistent with previous studies set in daily life, corroborating the notion that *why* one listens to music might be more relevant for stress reduction in daily life than the kind of music one listens to^30^. In this regard, we found evidence for differential effects of reasons for music listening between the two mental health conditions. Our results suggest that engaging in music listening for ‘distraction’, ‘acitvation’, and ‘no reason’ might be less beneficial for stress reduction in individuals with SSD compared to individuals with DEP. In DEP, rumination is a common and dysfunctional response to stressful events in daily life^49^. Thus, listening to music for the specific reason of ‘distraction’ might act as valuable stress-reduction strategy in individuals with DEP by diverting ruminative thoughts. In addition, music might facilitate behavioral activation (e.g., physical activity), which has been shown to be an evidence-based intervention for the treatment of DEP^50^. In individuals with SSD, ruminative thoughts typically focus more strongly on distress-causing bodily perceptions (e.g., pain catastrophizing) and they might try to avoid activating physical activities^13,17^. Thus, ‘distraction’ and ‘activation’ via music listening might not be as helpful for reducing subjective stress levels in individuals with SSD compared to individuals with DEP when music is self-administered in daily life. Previous research has emphasized that some individuals with mental health conditions might benefit from learning to use music in a more adaptive way for purposes of self-regulation^51^. Thus, more research is needed to elucidate whether reasons for music listening could be modified to further enhance the benefits of music in individuals suffering from SSD. Interestingly, having listened to music for ‘no reason’ was associated with lower sAA output, indicating a downregulation of ANS activity, for both groups. The answer category of ‘no reason’ might include a range of less target-oriented reasons, e.g., to increase pleasure through aesthetic experiences and/or to evoke positive memories or imagination^52^, that might exert particularly positive effects on autonomic arousal. Beyond motivations for music listening, future research should address further processes potentially explaining the effects of music listening on stress levels, for instance, whether a stress-reducing effect is due to cognitive mechanisms (e.g., cognitive reappraisal), induced emotions, or both, and how these processes might differ by mental health condition^53^.

Finally, we found evidence that music listening might mitigate somatic symptoms indirectly, mediated via a reduction in subjective stress levels. This indirect effect was observable even up to several hours after music listening. This crucial finding partly supports the biopsychological mediation model of music listening^23,37^ and indicates a central within-person mechanism translating the effects of music listening into reduced somatic symptoms. We did not detect any mediation effects of the relationship between music listening and somatic symptoms via HPA axis or ANS activity. However, rather than concluding that there is no such mediation, it should be kept in mind that the temporal associations between the effects of music on biological stress systems, and in turn, the effects of biological stress systems on subsequent somatic symptoms, might not have been covered by the time-contingent sampling design in the present study. In order to further elucidate the differential response patterns of the stress systems related to musical stimulation and their potential impact on somatic complaints, future ambulatory assessment (intervention) studies^54^ could be implemented that provide music particularly upon detection of high subjective and/or biological stress markers and repeatedly assess stress levels and somatic symptoms.

### Strengths and limitations

To the best of our knowledge, the present study is the first to investigate naturally occurring music listening behavior and its dynamic associations to somatic symptoms and stress markers in individuals suffering from SSD and DEP. Nevertheless, several limitations need to be critically acknowledged. First, self-reports of music listening referred to the time frame between the current and the previous data entry (i.e., comprising up to 3 to 4 h). In this regard, our findings are broadly comparable to previous studies with a similar design undertaken by our lab that have provided evidence for the beneficial effects of music listening on stress and pain parameters^30,33^. This corroborates the idea that the effects of music listening in daily life can be maintained over several hours. Nonetheless, we were not able to examine how music impacted psychobiological parameters *during* music listening, nor do the present findings allow conclusions on longer-term effects. Future studies could include event-based assessments in order to further unravel immediate as well as time-lagged effects of music listening on psychobiological stress markers and somatic complaints. Moreover, we cannot rule out that participants forgot to report music listening episodes, and we have no information on which music, exactly, participants listened to regarding genre or objective music characteristics. In order to expand on the present findings, music sensing applications could be implemented in future studies, as these can track such data without increasing participant burden. In addition, we cannot rule out that part of our results might also be explained by a placebo-like effect. One might argue that if a placebo-like effect was the main driver for our results, it would be difficult to explain why, for example, predominantly ‘happy’ music predicted lower somatic symptoms. Moreover, there is first evidence that the effects of music listening are not boosted by an expectancy induction, although expectancy-based placebo analgesia and music-induced analgesia might share some common psychobiological pathways^55^. Nonetheless, future research is needed to examine the role of potential placebo-like effects in the context of music listening. Furthermore, we only recruited women with medically not fully explained somatic symptoms. Therefore, replications in men and in individuals suffering from other conditions are necessary in order to determine to what extent the present findings might generalize to or differ from other populations. We did not include a healthy control group, as somatic symptoms are usually not observable on a daily basis in healthy individuals and thus, we did not consider healthy individuals appropriate for testing the main hypotheses of the study. Consequently, in our discussion of findings, we could only draw comparisons to previous ambulatory assessment studies undertaken with healthy individuals. An additional limitation concerns the lack of a formal a priori sample size calculation. Based on post-hoc power simulations (using the R-package “EMAtools”^56^), we achieved sufficient statistical power to detect medium and large effect sizes for the analyses combining SSD and DEP with music listening (yes/no) as predictor (data not shown). However, the total number of observations in the analyses with music characteristics and reasons for music listening was reduced to the number of reported music episodes. Moreover, particularly cross-level interactions require large sample sizes. Therefore, we cannot rule out an under-powering of our results (for an in-depth discussion of sample size and power in multilevel modeling see^57^) and conclude that a replication in larger-scaled studies is important. Finally, we want to emphasize that the present findings do not necessarily imply causality. As this study was observational in nature, we cannot rule out additional confounding variables or retrospective bias, even if the time delay between music listening and actual data entry was relatively short. However, we considered a temporal precedence in our approach (i.e. music listening was reported to have occurred before the assessment of stress markers and somatic complaints), and we included a comprehensive set of covariates. Thus, in our view, the present findings go beyond mere cross-sectional findings and therefore add substantially to the growing literature on the health-beneficial effects of music listening in everyday life^30–33,41,48^.

## Conclusion

Overall, music listening can be considered a beneficial self-management tool for the mitigation of somatic symptoms and stress reduction applicable in the daily life of women suffering from SSD and DEP. We were able to show that perceived music characteristics play an important modulating role in this regard, with music perceived as high in valence and low in arousal (i.e., happy and calming music) predicting lower somatic symptoms and ANS activity, respectively. Furthermore, we found preliminary evidence that why one listens to music might be of differential effectiveness for stress reduction, depending on mental health condition (or potentially associated characteristics e.g., trait emotion regulation), which warrant further investigation. In addition, our findings indicate that a reduction of subjective stress via music listening might benefit somatic symptoms for up to several hours later. The latter finding emphasizes the need to target stress-related mechanisms underlying the health-beneficial effects of music listening in future research. Findings from the present study are of practical relevance for practitioners considering music as a non-pharmacological complementary intervention for patients with somatic complaints. Future research on the health-beneficial effects of music listening could expand the current study by providing music at times when it is most needed (e.g., upon detection of high levels of stress or somatic complaints), adapting musical valence and arousal accordingly, incorporating objective measures of music listening and encouraging personalized motivations for music listening engagement.

## Methods

### Participants

Thirty women with SSD and 30 women with DEP (n = 28 with a current major depressive episode and n = 2 with dysthymia) were included in the study. We excluded one participant from each group due to very low compliance rates with the study protocol (i.e., more than 50% missing data), resulting in 29 women per group. We decided to include only individuals who self-identified as female due to sex differences in stress-related biomarkers^58^ and the higher prevalence rates of SSD and DEP among women^59^. For the diagnosis of SSD, a detailed diagnostic interview based on DSM-5 criteria was conducted^60,61^. For the diagnosis of DEP, the Structured Clinical Interview for DSM-IV was applied^62^. In order to achieve two distinct groups, individuals who met core criteria for both diagnoses concurrently were excluded. Furthermore, the following inclusion criteria were applied: no chronic physical illness that may (fully) explain the somatic symptoms, sufficient understanding of the German language, age between 18 and 65 years, body mass index (BMI) ≤ 30 kg/m^2^, regular menstrual cycle or postmenopausal for at least one year, no pregnancy or current breastfeeding, no other acute or unmedicated chronic conditions known to affect biological stress markers, no comorbid lifetime psychotic or bipolar disorder/borderline personality disorder/eating disorder or substance abuse within the past five years, no acute suicidality/self-harm behavior within the last six months, and no current psychotherapeutic treatment for the diagnosis of SSD or DEP.

### Procedure

Potential participants were identified from the waiting list of the Outpatient Clinic for Psychotherapy affiliated to the University of Marburg, Germany, or recruited through university mailing lists, online postings and flyers. Interested individuals were contacted by telephone and underwent a semi-structured interview conducted by trained research staff to check for inclusion and exclusion criteria. Eligible individuals were invited to the laboratory of the Department of Psychology, University of Marburg, Germany, where they completed psychometric online questionnaires via Unipark (Questback GmbH) and were instructed in the handling of an iPod® touch for data collection during the ambulatory assessment period. Furthermore, they received instructions on the correct collection and storage of saliva samples. The ambulatory assessment period started one day later and lasted for 14 consecutive days. Thereafter, participants returned to the laboratory to hand back the electronic device and the saliva samples and to complete a final set of questionnaires.

The Ethics Committee of the Department of Psychology at the University of Marburg, Germany, approved the study (2014-14k). All methods were performed in accordance with the Declaration of Helsinki. All participants provided written informed consent. Participants received a compensation of 80€ for complete study participation.

### Measures and materials

#### Demographic and psychometric questionnaires

Besides sociodemographic information, participants completed a detailed medical history. Moreover, two subscales from the Patient Health Questionnaire (PHQ) were used^63^. The PHQ-15 includes 13 items on impairment by specific somatic symptoms during the past four weeks, which are rated on a 3-point scale from 0 (‘not bothered at all’) to 2 (‘bothered a lot’). Two items that assess tiredness/low energy and sleep problems belonging to the PHQ-9 are usually included in the sum score of the PHQ-15. We excluded these two items from the PHQ-15 scale in order to better differentiate the scores from the PHQ-9 scale. The PHQ-9 covers impairment during the past two weeks by nine symptoms that are indicative of depressive disorders rated on a scale from 0 (‘not at all’) to 3 (‘almost all days’) (see Table 1). Finally, the revised version of the Music Preference Questionnaire, MPQ-R^64^, was applied to measure aspects related to habitual music behavior including importance of music in life assessed on a Likert scale from 1 (‘not at all important’) to 5 (‘very important’), the estimated daily duration of music listening (in minutes), and further music-related aspects (see online Supplemental Material, Table S1).

#### Ambulatory assessment

Participants were prompted to answer questions six times per day for 14 consecutive days via the application iDialogPad (Mutz, Cologne, Germany) installed on an iPod® touch. The first daily data entry was self-initiated upon awakening, followed by five prompts at 30 min after awakening, 11 a.m., 2 p.m., 6 p.m., and 9 p.m.. Since there were no questions on music listening on the first assessment of the day, only data from the five remaining daily prompts were used for analyses in the present study. From a maximum of 2030 possible observations per group, 6.4% (n = 129) of observations were missing (i.e., not responded to) in the SSD group and 9.4% (n = 191) in the DEP group. On the participant level, missing data ranged from 0 to 31.4%. Furthermore, data that were entered 2 h or later after the initial alarm were discarded, which applied to 2.6% (n = 53) of observations in the SSD group and 3.2% (n = 65) of observations in the DEP group.

##### Music listening behavior

At each measurement time point, participants were asked if they had deliberately listened to music since the last data entry (0 = no, 1 = yes). Thus, the item referred to the time span between the current and the previous data entry. If preceding music listening was reported, further questions on perceived music characteristics followed. Participants were asked to rate the valence of the music on a visual analog scale (VAS) ranging from 0 (‘sad’) to 100 (‘happy’) and to indicate the arousal of the music on a VAS ranging from 0 (‘relaxing’) to 100 (‘energizing’). Moreover, we asked participants to indicate their reasons for music listening. Specifically, we asked participants to indicate their reasons for music listening by choosing one or more of the following: ‘relaxation’, ‘activation’, ‘distraction’, ‘reducing boredom’, and ‘no reason’, which are considered the most frequently reported reasons for music listening^30^. Each reason was coded 0 (not selected) or 1 (selected).

##### Somatic symptoms

In line with state-of-the art recommendations^65^, participants were asked to indicate the momentary intensity of somatic symptoms by rating the statement “At the moment, my somatic complaints are intense” on a VAS ranging from 0 (‘not at all’) to 100 (‘strongest imaginable’), as well as the impairment by somatic symptoms by rating the statement “At the moment, I feel impaired by somatic complaints” on a VAS ranging from 0 (‘not at all’) to 100 (‘very much’).

##### Subjective stress

In order to keep participant burden as low as possible, a one-item measure was used to assess momentary subjective stress. This approach was shown to be valid and reliable^66^. Participants responded to the item “At the moment, I feel stressed” on a 5-point Likert scale ranging from 0 (‘not at all’) to 4 (‘very much’).

##### Biological stress markers

Saliva samples for the analysis of salivary cortisol (sCort) as an endocrine marker of HPA axis activity and salivary alpha-amylase (sAA) as an autonomic marker indicating ANS activity were taken at each measurement time point^67^. Participants were instructed to accumulate unstimulated saliva in the oral cavity for two minutes, which was indicated by a countdown within the app, and to subsequently transfer the saliva into polypropylene tubes via a straw (SaliCap®, IBL, Hamburg, Germany). Participants were asked to store the collected saliva samples in their freezer or refrigerator at home during the ambulatory assessment period. Biochemical analyses were conducted at the Biochemical Laboratory, University of Marburg, Germany. Samples were kept frozen at − 20 °C, and were thawed and centrifuged on the day of analysis. Levels of sCort were measured using a commercially available enzyme-linked immunoassay (IBL, Hamburg, Germany). sAA activity was determined from saliva samples using a kinetic colorimetric test and reagents from Roche (Roche Diagnostics, Mannheim, Germany). Since previous research indicates that salivary flow rate might be associated with the concentration of alpha-amylase in accumulated saliva^67^, we adjusted sAA activity for salivary flow rate resulting in sAA output (U/min)^68^. Both sCort and sAA output were log-transformed due to skewed distributions using the formula ln(*x*) + 10. Intra- and interassay variances for sCort were ≤ 10%. For sAA, interassay variance was < 13% and intraassay variance was < 10%.

##### Biobehavioral control variables

Several biobehavioral control variables that have been shown to affect somatic symptoms and stress markers in previous studies were assessed at each measurement time point for statistical control^67,69^. These included time since awakening (in minutes), additional intake of medication since the last data entry (0 = no, 1 = yes), sleep quality reported at awakening on a VAS from 0 (‘sleep was not at all restful’) to 100 (‘sleep was very restful’), as well as physical activity in the past hour ranging from 0 (‘not at all active’) to 100 (‘very active’). Due to the potential impact on biological stress markers, consumption of food (0 = no, 1 = yes) and beverages (0 = no, 1 = yes) in the past hour was also assessed.

### Statistical analyses

For the analyses of hypotheses 1 and 2, we specified multilevel models using the statistical software HLM 7.03 (Scientific Software International Inc., Lincolnwood, USA) with observations (level 1) nested within participants (level 2). The intraclass correlation coefficients (ICCs) indicated a high variability between individuals on all outcome variables, substantiating the nested structure of the data (see Table 2). Exemplary equations and the detailed rationale for inclusion of control variables on both levels are provided in the Supplemental Material available online.

We specified separate multilevel models for each outcome variable (i.e., intensity of somatic symptoms, impairment by somatic symptoms, subjective stress, sCort, sAA). In accordance with previous recommendations^70^, we first computed a random-intercept model including covariates only, combining data from both groups (= control model). On level 1, time since awakening, irregular medication intake, physical activity, and sleep quality were included as control variables in all models. Consumption of food and beverages in the past hour were additionally included as level-1 covariates in models with sCort and sAA output as outcomes, respectively. On level 2 (person level), the intercept was modeled as a function of mental health condition (SSD = 1, DEP = 0), age, BMI, intake of antidepressant and/or pain medication, PHQ-15 score, PHQ-9 score, and total number of music listening episodes per individual. In the next step, we added music listening (yes = 1, no = 0) as predictor of interest and investigated the main effect on the respective outcome variable. Models included a random slope for music listening, thus, the association between music listening and the respective outcome was allowed to vary between individuals. In the third step, we examined whether the association between music listening and the outcome was moderated by mental health condition via inclusion of a cross-level interaction term (music listening x condition). The same three-step procedure was undertaken when investigating music characteristics (including musical valence and musical arousal simultaneously) and reasons for music listening (including all reasons simultaneously). The total number of observations was reduced in models with music characteristics and reasons for music listening since these data were only available when music listening was reported. Thus, the final number of included observations in the models ranged from 552 to 3584. Model estimation was performed using restricted maximum likelihood with listwise deletion in the case of missing values. All level-1 predictors (except for time since awakening and sleep quality) were group-mean centered to disentangle within-person from between-person effects^71^. Level-2 variables measured on continuous scales were grand-mean centered and dichotomous level-2 variables remained uncentered. As a measure of effect size, we report Pseudo-*R*^*2*^, indicating the reduction in residual level-1 variance in the outcome via inclusion of the predictor(s) of interest to the control model, which can be calculated using the formula (*σ*^*2*^ control model − *σ*^*2*^ final model)/*σ*^*2*^ control model)^72^. In addition, model comparisons between control model and subsequent models were undertaken by means of *Ӽ*^*2*^-statistics using full maximum likelihood estimation, which compares the reduction in deviance as a measure of model fit. For all analyses, we report unstandardized coefficients (UC) and consider *p*-values < 0.05 as significant.

When testing hypothesis 3, we adhered to guidelines for multilevel mediation analyses^73^. Since music-related predictors, stress markers (mediators), and somatic symptoms (outcomes) were all measured on level 1, we specified 1–1–1 multilevel mediation models using the macro Mlmed for SPSS^74^. We initially tested multilevel mediation models with random slopes (i.e. variation across individuals) for all 1–1–1 associations. In case of non-significant random slopes or non-convergence, we dropped random slopes, investigated model fit indices (Aikake’s Information Criteria, AIC; Bayesian Information Criteria, BIC) and preferred the model that provided a better fit. We first tested mediation models in which all variables were assessed concurrently. A temporal distance between music-related predictors and stress markers is given in these models (i.e., music listening preceded stress markers), but stress markers and somatic symptoms reflected both momentary levels at the same time point of assessment. Thus, in order to consider temporal precedence between mediators and outcomes as an important conceptual aspect of mediation^75^, we additionally tested models in which somatic symptoms from the subsequent measurement time point were used as outcomes (i.e., time-lagged). We used restricted maximum likelihood for model estimation and 10,000 samples to determine 95% Monte Carlo confidence intervals (MCCI) for indirect effects.

## Supplementary Information

Supplementary Information.

## Data Availability

The datasets generated during and analysed during the current study are available from the corresponding author on reasonable request.
