# Supplementary material for: The effects of music listening on somatic symptoms and stress markers in the everyday life of women with somatic complaints and depression
Source: Sci Rep. 2021 Dec 15;11:24062. doi: 10.1038/s41598-021-03374-w (PMC8674261; doi:10.1038/s41598-021-03374-w)
Supplement: Supplementary file 1 — Supplementary Information. [file 41598_2021_3374_MOESM1_ESM.docx]

**Supplemental Material**

**The effects of music listening on somatic symptoms and stress markers in the everyday life of women with somatic complaints and depression**

by Anja C. Feneberg, Ricarda Mewes, Johanna M. Doerr, Urs M. Nater

**Table of Contents**

[Exemplary model equations for hypotheses 1 and 2 2](#_Toc64207455)

[Rationale for inclusion of control variables in multilevel models 5](#_Toc64207456)

[Table S1 6](#_Toc64207457)

[Table S2 8](#_Toc64207458)

[Table S3 9](#_Toc64207459)

[References 10](#_Toc64207460)

# **Exemplary model equations for hypotheses 1 and 2**

**Key**

#### **Level 1 variables**

DRINK = Consumption of beverages in the hour before data entry (1 = yes, 0 = no)

FOOD = Consumption of food in the hour before data entry (1 = yes, 0 = no)

INTSOMSY = Momentary intensity of somatic symptoms

SAALN = Salivary alpha-amylase output (U/min)

MEDI = Medication intake since the last data entry

ML = Music listening since previous data entry (yes = 1, no = 0)

MACTIV = Activation (reason for music listening; yes = 1, no = 0)

MDISTR = Distraction (reason for music listening; yes = 1, no = 0)

MREDBOR = Reducing boredom (reason for music listening; yes = 1, no = 0)

MRELAX = Relaxation (reason for music listening; yes = 1, no = 0)

MNOREA = No reason (reason for music listening; yes = 1, no = 0)

M_VALEN = Perceived musical valence

M_AROUS = Perceived musical arousal

PA = Physical activity in the hour before data entry

SLEEP = Sleep quality reported at waking

TSA_MIN = Time since awakening in minutes

#### **Level 2 variables**

BMI = Body Mass Index (in kg/m²)

GROUP = Dummy for group (SSD = 1, DEP = 0)

M_Antidep = Intake of antidepressant medication (yes = 1, no = 0)

M_Pain = Intake of pain medication

MLCOUNT = Overall number of music listening episodes

PHQ15 = severity of somatic symptoms

PHQ9 = severity of depressive symptoms

***Example 1***. Intensity of somatic symptoms as a function of music listening, the cross-level interaction music listening x group, and covariates

#### **Level-1 Model**

*INTSOMSYy_ti_* = *π_0i_* + *π_1i_**(*ML_ti_*) + *π_2i_**(*TSA_MIN_ti_*) + *π_3i_**(*PA_ti_*) + *π_4i_**(*MEDI_ti_*) + *π_5i_**(*SLEEP_ti_*) + *e_ti_*

#### **Level-2 Model**

*π_0i_* = *β_00_* + *β_01_**(*GROUP_i_*) + *β_02_**(*AGE_i_*) + *β_03_**(*BMI_i_*) + *β_04_**(*PHQ15_i_*) + *β_05_**(*PHQ9_i_*) + *β_06_**(*MLCOUNT_i_*) + *β_07_**(*M_ANTDEP_i_*) + *β_08_**(*M_PAIN_i_*) + *r_0i_*
    *π_1i_* = *β_10_* + *β_11_**(*GROUP_i_*) + *r_1i_*
    *π_2i_* = *β_20_*
    *π_3i_* = *β_30_*
    *π_4i_* = *β_40_*
    *π_5i_* = *β_50_*

ML PA MEDI have been centered around the group mean.

Age BMI PHQ15 PHQ9 MLCOUNT have been centered around the grand mean.

#### **Mixed Model**

*INTSOMSY_ti_* = *β_00_* + *β_01_***GROUP_i_* + *β_02_***AGE_i_* + *β_03_***BMI_i_* + *β_04_***PHQ15_i_* + *β_05_***PHQ9_i_* + *β_06_***ML_COUNT_i_* + *β_07_***M_ANTDEP_i_* + *β_08_***M_PAIN_i_* + *β_10_***M_ti_* + *β_11_***GROUP_i_***ML_ti_* + *β_20_***TSA_MIN_ti_* + *β_30_***PA_ti_* + *β_40_***MEDI_ti_* + *β_50_***SLEEP_ti_* + *r_0i_* + *r_1i_***ML_ti_* + *e_ti_*

***Example 2***. Intensity of somatic symptoms as a function of reasons for music listening and covariates

#### **Level-1 Model**

*INTSOMSY_ti_* = *π_0i_* + *π_1i_**(*MRELAX_ti_*) + *π_2i_**(*MACTIV_ti_*) + *π_3i_**(*MDISTR_ti_*) + *π_4i_**(*MREDBOR_ti_*) + *π_5i_**(*MNOREAS_ti_*) + *π_6i_**(*TSA_MIN_ti_*) + *π_7i_**(*PA_ti_*) + *π_8i_**(*MEDI_ti_*) + *π_9i_**(*SLEEP_ti_*) + *e_ti_*

#### **Level-2 Model**

*π_0i_* = *β_00_* + *β_01_**(*GROUP_i_*) + *β_02_**(*AGE_i_*) + *β_03_**(*BMI_i_*) + *β_04_**(*PHQ15_i_*) + *β_05_**(*PHQ9_i_*) + *β_06_**(*MLCOUNT_i_*) + *β_07_**(*M_ANTDEP_i_*) + *β_08_**(*M_PAIN_i_*) + *r_0i_*
    *π_1i_* = *β_10_* + *r_1i_*
    *π_2i_* = *β_20_* + *r_2i_*
    *π_3i_* = *β_30_* + *r_3i_*
    *π_4i_* = *β_40_* + *r_4i_*
    *π_5i_* = *β_50_* + *r_5i_*
    *π_6i_* = *β_60_*
    *π_7i_* = *β_70_*
    *π_8i_* = *β_80_*
    *π_9i_* = *β_90_*

MRELAX MACTIV MDISTR MREDBOR MNOREAS PA MEDI have been centered around the group mean.

AGE BMI PHQ15 PHQ9 MLCOUNT have been centered around the grand mean.

#### **Mixed Model**

*STRESS_ti_* = *β_00_* + *β_01_***GROUP_i_* + *β_02_***AGE_i_* + *β_03_***BMI_i_* + *β_04_***PHQ15_i_* + *β_05_***PHQ9_i_* + *β_06_***MLCOUNT_i_* + *β_07_***M_ANTDEP_i_* + *β_08_***M_PAIN_i_* + *β_10_***RELAX_ti_* + *β_20_***ACTIV_ti_* + *β_30_***DISTR_ti_* + *β_40_***REDBOR_ti_* + *β_50_***NOREAS_ti_* + *β_60_***TSA_MIN_ti_* + *β_70_***PA_ti_* + *β_80_***MEDI_ti_* + *β_90_***SLEEP_ti_* + *r_0i_* + *r_1i_***RELAX_ti_*  + *r_2i_***ACTIV_ti_*  + *r_3i_***DISTR_ti_* + *r_4i_***REDBOR_ti_*  + *r_5i_***NOREAS_ti_* + *e_ti_*

***Example 3.*** Salivary alpha-amylase output (U/min)^1^ as a function of music characteristics (valence, arousal), cross-level interactions (valence x group, arousal x group), and covariates

#### **Level-1 Model**

*SAALN_ti_* = *π_0i_* + *π_1i_**(*M_VALEN_ti_*) + *π_2i_**(*M_AROUS_ti_*) + *π_3i_**(*TSA_MIN_ti_*) + *π_4i_**(*PA_ti_*) + *π_5i_**(*MEDI_ti_*) + *π_6i_**(*FOOD_ti_*) + *π_7i_**(*DRINK_ti_*) + *π_8i_**(*SLEEP_ti_*) + *e_ti_*

#### **Level-2 Model**

*π_0i_* = *β_00_* + *β_01_**(*GROUP_i_*) + *β_02_**(*AGE_i_*) + *β_03_**(*BMI_i_*) + *β_04_**(*PHQ15_i_*) + *β_05_**(*PHQ9_i_*) + *β_06_**(*MLCOUNT_i_*) + *β_07_**(*M_ANTDEP_i_*) + *β_08_**(*M_PAIN_i_*) + *r_0i_*
    *π_1i_* = *β_10_* + *β_11_**(*GROUP_i_*) + *r_1i_*
    *π_2i_* = *β_20_* + *β_21_**(*GROUP_i_*) + *r_2i_*
    *π_3i_* = *β_30_*
    *π_4i_* = *β_40_*
    *π_5i_* = *β_50_*
    *π_6i_* = *β_60_*
    *π_7i_* = *β_70_*
    *π_8i_* = *β_80_*

M_VALEN M_AROUS PA MEDI FOOD DRINK have been centered around the group mean.

AGE BMI PHQ15 PHQ9 MLCOUNT have been centered around the grand mean.

#### **Mixed Model**

*SAALN_ti_* = *β_00_* + *β_01_***GROUP_i_* + *β_02_***AGE_i_* + *β_03_***BMI_i_* + *β_04_***PHQ15_i_* + *β_05_***PHQ9_i_* + *β_06_***MLCOUNT_i_* + *β_07_***M_ANTDEP_i_* + *β_08_***M_PAIN_i_* + *β_10_***MVALEN_ti_* + *β_11_***GROUP_i_***M_VALEN_ti_* + *β_20_***M_AROUS_ti_* + *β_21_***GROUP_i_***MAROUS_ti_* + *β_30_***TSA_MIN_ti_* + *β_40_***PA_ti_* + *β_50_***MEDI_ti_* + *β_60_***FOOD_ti_* + *β_70_***DRINK_ti_* + *β_80_***SLEEP_ti_* + *r_0i_* + *r_1i_***M_VALEN_ti_*  + *r_2i_***M_AROUS_ti_* + *e_ti_*

^1^transformed values using the formula ln(*x*)+10 were used in the analyses in order to meet the assumptions of multilevel models (e.g., normally distributed residuals)

# **Rationale for inclusion of control variables in multilevel models**

**Level-1 covariates**

Time since awakening (in minutes) was included in all models in order to control for the diurnal variations of the outcome variables (Schlotz, 2019; Singer & Willett, 2003). In addition, irregular medication intake since the last data entry, physical activity, and sleep quality were considered potentially confounding variables on level 1 as indicated by previous research on somatic symptoms and/or stress parameters in clinical populations (Fischer et al., 2016; Klaus, Fischer, Doerr, Nater, & Mewes, 2017). Models examining biological stress markers as outcomes additionally included consumption of food and beverages in the past hour, since these have been shown to impact secretion of both biomarkers (Strahler et al., 2017).

**Level-2 covariates**

On level 2, group (SSD vs. DEP, with DEP as the reference group), age, BMI, and intake of antidepressant and pain medication were included since these were considered of primary relevance for their potential impact on the outcome variables on the person-level (Klaus, Fischer, Doerr, Nater, & Mewes, 2017; Strahler et al., 2017; Huffziger et al., 2013). In addition, two subscales from the Patient Health Questionnaire (PHQ; Löwe, Spitzer, Zipfel, & Herzog, 2002), the PHQ-15 and PHQ-9, were included to control for severity of somatic and depressive symptoms in the weeks before the study started. Two items that assess tiredness/low energy and sleep problems belonging to the PHQ-9 are usually included in the sum score of the PHQ-15. We excluded these two items from the PHQ-15 scale in order to better differentiate the scores from the PHQ-9 scale. Finally, since the data revealed a substantive variation in music listening episodes between individuals and in line with previous research (Linnemann et al., 2015), we included total number of music listening episodes during the ambulatory assessment period as level-2 covariate.

| Table S1*Habitual music behaviour by mental health condition* | | | | | | | | |
| --- | --- | --- | --- | --- | --- | --- | --- | --- |
|  |  | SSD |  | DEP |  | Test parameter | df | *p* |
|  |  | N = 29  mean±SD |  | N = 29  mean±SD |  |  |  |  |
| Daily duration of music listening (min)^a^ |  | 97.6±87.3 |  | 141.4±120.2 |  | *t* = 1.60 | 54 | .13 |
| Importance of music in life^b^ |  | 4.3±0.8 |  | 4.3±0.9 |  | *t*<0.01 | 56 | >.99 |
| *Preferences for music styles*^c^ |  |  |  |  |  |  |  |  |
| Pop |  | 2.9±1.5 |  | 3.2±1.5 |  | *t* = 0.72 | 56 | .48 |
| Rock |  | 3.7±1.3 |  | 3.9±1.3 |  | *t* = 0.60 | 56 | .55 |
| Hip Hop |  | **1.9±1.2** |  | **2.6±1.2** |  | ***t* = 2.30** | **56** | **.025** |
| Latin |  | 2.3±1.3 |  | 2.1±1.3 |  | *t* = -0.50 | 56 | .62 |
| Soul/Funk |  | 2.7±1.3 |  | 2.6±1.3 |  | *t* = 1.34 | 56 | .19 |
| Hard Rock |  | 2,1±1.3 |  | 2.3±1.4 |  | *t* = 0.68 | 56 | .50 |
| Electronic Music |  | 2.3±1.4 |  | 2.7±1,2 |  | *t* = 1.10 | 56 | .28 |
| New Age |  | 2.2±1.2 |  | 2.2±1.4 |  | *t* = -0.10 | 56 | .92 |
| Folk Music |  | 1.6±0.9 |  | 1.8±1.2 |  | *t* = 0.62 | 56 | .54 |
| Classic Music |  | 3.1±1.5 |  | 3.0±1.3 |  | *t* = -0.38 | 56 | .70 |
| Jazz/Blues |  | 2.8±1.6 |  | 2.3±1.3 |  | *t* = 1.45 | 56 | .15 |
| *Frequency of music-listening reasons*^d^ |  |  |  |  |  |  |  |  |
| Relaxation |  | 4.0±1.1 |  | 3.7±0.9 |  | *t* = 1.04 | 56 | .30 |
| Activation |  | 4.4±0.9 |  | 4.1±1.2 |  | *t* = -0.84 | 56 | .40 |
| Distraction |  | 3.5±1.4 |  | 3.5±1.3 |  | *t* < 0.01 | 56 | >.99 |
| To reduce aggression |  | 2.8±1.5 |  | 2.6±1.5 |  | *t* = -0.45 | 56 | .66 |
| To work better |  | 3.2±1.4 |  | 2.8±1.5 |  | *t* = -0.98 | 56 | .33 |
| To evoke certain feelings |  | 3.4±1.5 |  | 3.2±1.6 |  | *t* = -0.52 | 56 | .61 |
| To increase certain feelings |  | 3.3±1.6 |  | 3.2±1.5 |  | *t* = -0.25 | 56 | .80 |
| Against boredom |  | 2.8±1.6 |  | 3.2±1.4 |  | *t* = 1.07 | 56 | .29 |
| Against loneliness |  | 2.5±1.6 |  | 2.8±1.4 |  | *t* = 0.77 | 56 | .44 |
| Because of the music |  | **4.2±1.2** |  | **3.4±1.7** |  | ***t* = -2.10** | **56** | **.040** |
| *Frequency of music-listening occasions*^d^ |  |  |  |  |  |  |  |  |
| Disco/Club |  | 2.8±1.3 |  | 2.7±1.3 |  | *t* = -0.41 | 56 | .69 |
| Techno Party |  | 1.7±1.2 |  | 1.8±1.2 |  | *t* = 0.44 | 56 | .66 |
| Concerts (Rock/Pop) |  | 2.5±1.3 |  | 2.4±1.3 |  | *t* = -0.20 | 56 | .84 |
| Concerts (Classical/Opera) |  | 2.2±1.3 |  | 1.7±1.1 |  | *t* = -1.39 | 56 | .17 |
| Background activity |  | 4.2±1.3 |  | 4.4±1.1 |  | *t* = 0.33 | 56 | .74 |
| ***TABLE*** ***S1****. continued* |  |  |  |  |  |  |  |  |
|  |  |  |  |  |  |  |  |  |
|  |  | SSD |  | DEP |  | Test parameter | df | *p* |
|  |  | N = 29  mean±SD |  | N = 29  mean±SD |  |  |  |  |
| Music Making (e.g., singing) |  | 2.5±1.6 |  | 2.7±1.4 |  | *t* = 0.70 | 56 | .49 |
| When being alone |  | 4.1±0.9 |  | 4.2±1.0 |  | *t* = 0.41 | 56 | .68 |
| When being with friends |  | 3.4±1.1 |  | 3.5±1.4 |  | *t* = 0.21 | 56 | .84 |
| *Music-induced chills* |  |  |  |  |  |  |  |  |
| Frequency of music-induced chills^e^ |  | 2.7±1.0 |  | 3.0±1.1 |  | *t* = 1.14 | 56 | .26 |
| Intensity of music-induced chills^f^ |  | 3.2±1.1 |  | 3.3±0.6 |  | *t* = 0.12 | 49 | .90 |
| *Active music making* |  | n (%) |  | n (%) |  |  |  |  |
| Currently playing an instrument |  | 6 (20.7%) |  | 7 (24.1%) |  | *Ӽ²* = 0.10 | 1 | .75 |
| Currently singing in a choir |  | 3 (10.3%) |  | 1 (3.5%) |  | *Ӽ²* = 1.10 | 1 | .30 |
| Playing an instrument in the past |  | 12 (41.4%) |  | 9 (31.0%) |  | *Ӽ²* = 0.38 | 1 | .53 |
| Singing in a choir in the past |  | 6 (20.7%) |  | 8 (27.6%) |  | *Ӽ²* = 0.76 | 1 | .38 |

All data assessed via the revised version of the Music Preference Questionnaire (Nater, Krebs, & Ehlert, 2005), (available on <https://www.musicandhealthlab.com/publications/>). DEP = Depressive disorders. SSD = Somatic symptom disorder. Significant estimates (*p* < .05) are marked in bold.

^a^One individual in the SSD group was excluded due to implausible reports, one individual in the DEP group did not provide information.

^b^Answered on a Likert-scale ranging from 1 (‘not at all important’) to 5 (‘very important’).

^c^Answered on a Likert scale ranging from 1 (‘not at all’) to 5 (‘very much’) per music style.

^d^Frequency per reason/occasion indicated on a Likert scale ranging from 1 (‘never’) to 5 (‘very often’).

^e^Frequency of chills experiences related to music listening indicated on a Likert scale ranging from 1 (‘never’) to 5 (‘almost every time’).

^f^Values are based on 25 individuals in the SSD group and 26 individuals in the DEP group that reported experiencing chills at least 2 (‘seldomly’).

| Table S2*Multilevel models for somatic symptoms predicted by music characteristics and covariates* | | | | | | | | | |
| --- | --- | --- | --- | --- | --- | --- | --- | --- | --- |
|  | **Model 1a)** Intensity of somatic symptoms | | | |  | **Model 1b)** Impairment by somatic symptoms | | | |
| *Fixed Effects* | UC | SE | df | *p* |  | UC | SE | df | *p* |
| *Level 2* |  |  |  |  |  |  |  |  |  |
| Intercept level 2 | **12.92** | **4.23** | **48** | **.004** |  | **15.56** | **4.31** | **48** | **<.001** |
| Group^a^ | 8.70 | 6.44 | 48 | .18 |  | 2.35 | 6.22 | 48 | .71 |
| Age | 0.14 | 0.19 | 48 | .47 |  | 0.20 | 0.19 | 48 | .31 |
| BMI | 0.06 | 0.61 | 48 | .92 |  | 0.17 | 0.62 | 48 | .79 |
| PHQ-15 | **1.95** | **0.54** | **48** | **<.001** |  | **1.96** | **0.52** | **48** | **<.001** |
| PHQ-9 | 0.02 | 0.52 | 48 | .97 |  | -0.22 | 0.49 | 48 | .66 |
| Number of music episodes | 0.01 | 0.27 | 48 | .98 |  | 0.07 | 0.26 | 48 | .80 |
| Antidepressant medication | 10.12 | 7.17 | 48 | .17 |  | 7.56 | 7.42 | 48 | .31 |
| Pain medication | -1.20 | 5.28 | 48 | .82 |  | 1.71 | 5.21 | 48 | .74 |
| *Level 1* |  |  |  |  |  |  |  |  |  |
| Musical valence | **-0.14** | **0.05** | **56** | **.012** |  | **-0.14** | **0.06** | **56** | **.012** |
| Musical arousal | -0.00 | 0.04 | 56 | .96 |  | 0.00 | 0.04 | 56 | .84 |
| Time since awakening | 0.00 | 0.00 | 413 | .66 |  | 0.00 | 0.00 | 413 | .89 |
| Physical activity | 0.02 | 0.04 | 413 | .49 |  | 0.03 | 0.03 | 413 | .36 |
| Medication intake | **15.20** | **3.54** | **413** | **<.001** |  | **20.85** | **3.87** | **413** | **<.001** |
| Sleep quality | 0.05 | 0.03 | 413 | .12 |  | 0.03 | 0.03 | 413 | .34 |
| *Random effects* | VC | SD | *χ^2^* (df) | *p* |  | VC | SD | χ2 (df) | *p* |
| Intercept level 1 | **137.23** | **11.72** | **220.21 (39)** | **<.001** |  | **152.72** | **12.36** | **214.77 (39)** | **<.001** |
| Musical valence | 0.02 | 0.14 | 54.14 (47) | .22 |  | 0.01 | 0.12 | 61.16 (47) | .080 |
| Musical arousal | 0.00 | 0.02 | 33.75 (47) | >.50 |  | 0.00 | 0.07 | 51.42 (47) | .30 |
| Residual | 287.17 | 16.95 |  |  |  | 344.86 | 18.57 |  |  |

UC = unstandardized coefficient. SE = standard error. df = degrees of freedom. VC = variance component. SD = standard deviation. PHQ-15 = somatic symptom subscale from the Patient Health Questionnaire. PHQ-9 = depression subscale from the Patient Health Questionnaire. Significant estimates (*p* < .05) are marked in bold. ^a^Group was coded 0 for DEP and 1 for SSD.

| Table S3*Multilevel model for subjective stress predicted by reasons for music listening interacted with mental health condition* | | | | |
| --- | --- | --- | --- | --- |
| *Fixed Effects* | UC | SE | df | *p* |
| *Level 2* |  |  |  |  |
| Intercept level-2 | **2.37** | **0.18** | **48** | **<.001** |
| Group^a^ | -0.40 | 0.25 | 48 | .12 |
| Age | 0.01 | 0.01 | 48 | .13 |
| BMI | 0.02 | 0.02 | 48 | .37 |
| PHQ 15 | 0.04 | 0.02 | 48 | .059 |
| PHQ 9 | 0.02 | 0.02 | 48 | .38 |
| Number of music episodes | -0.01 | 0.01 | 48 | .66 |
| Antidepressant medication | -0.35 | 0.29 | 48 | .24 |
| Pain medication | 0.19 | 0.21 | 48 | .36 |
| *Level 1* |  |  |  |  |
| Relaxation | -0.07 | 0.18 | 55 | .69 |
| Relaxation x group | 0.38 | 0.27 | 55 | .17 |
| Activation | -0.32 | 0.17 | 55 | .062 |
| Activation x group | **0.66** | **0.28** | **55** | **.020** |
| Distraction | -0.10 | 0.15 | 55 | .53 |
| Distraction x group | **0.61** | **0.25** | **55** | **.017** |
| Reducing boredom | -0.27 | 0.16 | 55 | .10 |
| Reducing boredom x group | 0.44 | 0.28 | 55 | .12 |
| No reason | **-0.57** | **0.27** | **55** | **.040** |
| No reason x group | **0.92** | **0.45** | **55** | **.046** |
| Time since awakening | **-0.00** | **0.00** | **242** | **<.001** |
| Physical activity | 0.00 | 0.00 | 242 | .97 |
| Medication intake | -0.19 | 0.19 | 242 | .33 |
| Sleep quality | **-0.00** | **0.00** | **242** | **.005** |
| *Random effects* | VC | SD | χ^2^ (df) | *p* |
| Intercept level-1 | 0.19 | 0.43 | 47.25 (0) | >.50 |
| Relaxation | 0.07 | 0.27 | 6.89 (7) | >.50 |
| Activation | 0.01 | 0.10 | 6.55 (7) | >.50 |
| Distraction | 0.02 | 0.14 | 2.61 (7) | >.50 |
| Reducing boredom | 0.04 | 0.20 | 3.58 (7) | >.50 |
| No reason | 0.44 | 0.66 | 13.61 (7) | .058 |
| Residual | 0.80 | 0.90 |  |  |

UC = unstandardized coefficient. SE = standard error. df = degrees of freedom. VC = variance component. SD = standard deviation. PHQ-15 = somatic symptom subscale from the Patient Health Questionnaire. PHQ-9 = depression subscale from the Patient Health Questionnaire. Significant estimates (*p* < .05) are marked in bold. ^a^Group was coded 0 for DEP and 1 for SSD.

**References**

Fischer, S., Doerr, J. M., Strahler, J., Mewes, R., Thieme, K., & Nater, U. M. (2016). Stress exacerbates pain in the everyday lives of women with fibromyalgia syndrome—The role of cortisol and alpha-amylase. *Psychoneuroendocrinology*, *63*, 68–77. <https://doi.org/10.1016/j.psyneuen.2015.09.018>

Huffziger, S., Ebner-Priemer, U., Zamoscik, V., Reinhard, I., Kirsch, P., &, Kuehner, C., (2013). Effects of mood and rumination on cortisol levels in daily life: an ambulatory assessment study in remitted depressed patients and healthy controls. *Psychoneuroendocrinology, 38*, 2258–2267. <https://doi.org/10.1016/j.psyneuen.2013.04.014>

Klaus, K., Fischer, S., Doerr, J.M., Nater, U. M., & Mewes, R. (2017). Classifying Fibromyalgia Syndrome as a Mental Disorder?-An Ambulatory Assessment Study. *International Journal of Behavioral Medicine, 24*, 230–238. <https://doi.org/10.1007/s12529-016-9603-6>

Linnemann, A., Kappert, M. B., Fischer, S., Doerr, J. M., Strahler, J., & Nater, U. M. (2015). The effects of music listening on pain and stress in the daily life of patients with fibromyalgia syndrome. *Frontiers in Human Neuroscience, 9*, 434. <https://doi.org/10.3389/fnhum.2015.00434>

Löwe, B., Spitzer, R. L., Zipfel, S., & Herzog, W. (2002). *Gesundheitsfragebogen für Patienten (PHQ D). Komplettversion und Kurzform*. Testmappe mit Manual, Fragebögen, Schablonen. Karlsruhe: Pfizer.

Nater, U. M., Krebs, M., & Ehlert, U. (2005). Sensation Seeking, Music Preference, and Psychophysiological Reactivity to Music. *Musicae Scientiae*, *9*(2), 239–254. <https://doi.org/10.1177/102986490500900205>

Schlotz, W., 2019. Investigating associations between momentary stress and cortisol in daily life: What have we learned so far? *Psychoneuroendocrinology, 105*, 105–116. <https://doi.org/10.1016/j.psyneuen.2018.11.038>

Singer, J. D., & Willett, J. B. (2003). *Applied longitudinal data analysis: Modeling change and event occurrence.* Oxford: Oxford Univ. Press.

Strahler, J., Skoluda, N., Kappert, M. B., & Nater, U. M. (2017). Simultaneous measurement of salivary cortisol and alpha-amylase: Application and recommendations. *Neuroscience and Biobehavioral Reviews*, *83*, 657–677. <https://doi.org/10.1016/j.neubiorev.2017.08.015>
